# Supplementary figures and images for: Neuron populations use variable combinations of short-term feedback mechanisms to stabilize firing rate
Source: PLoS Biol. 2023 Jan 23;21(1):e3001971. doi: 10.1371/journal.pbio.3001971 (PMC9894548; doi:10.1371/journal.pbio.3001971)

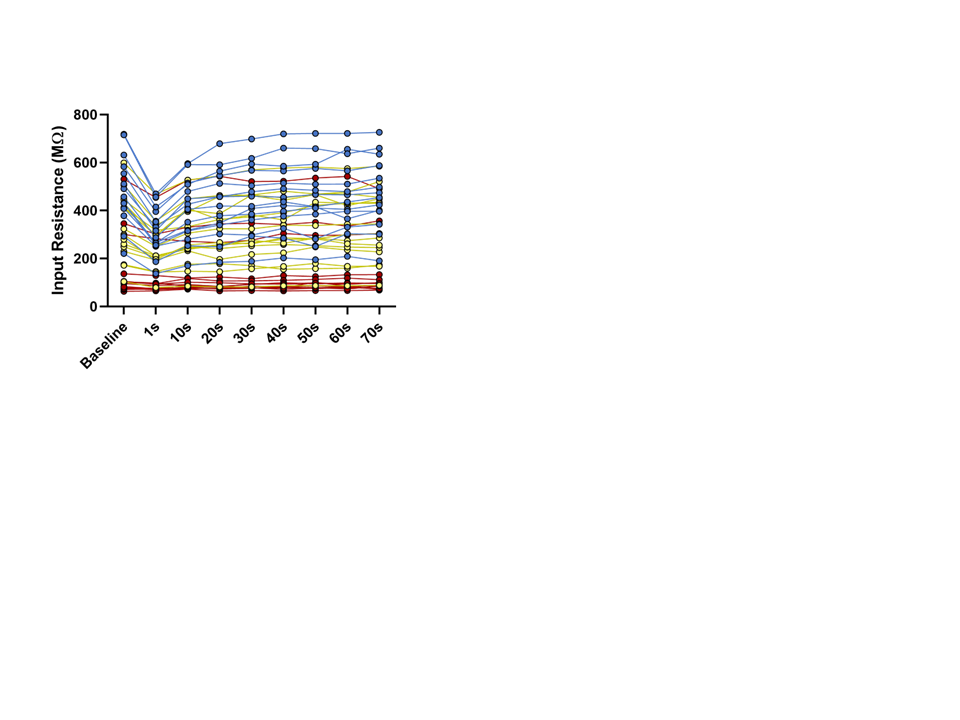

Supplement: S1 Fig — Data shown here are the same as shown in Fig 1C, but instead plotted as absolute rather than relative Rin. Red represents the smallest 1/3 of Rin changes in the dataset, green represents the middle 1/3, and blue shows the largest Rin drops in the dataset. The data underlying this figure can be found in S1 Data. (TIF) [file pbio.3001971.s001.tif]

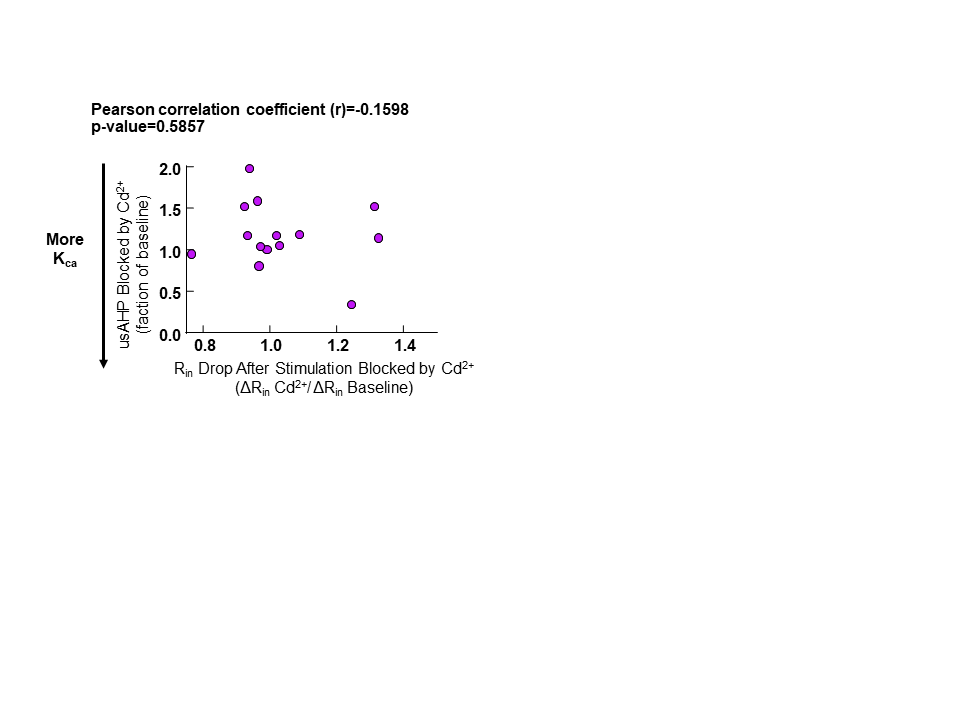

Supplement: S2 Fig — The data underlying this figure can be found in S1 Data. (TIF) [file pbio.3001971.s002.tif]

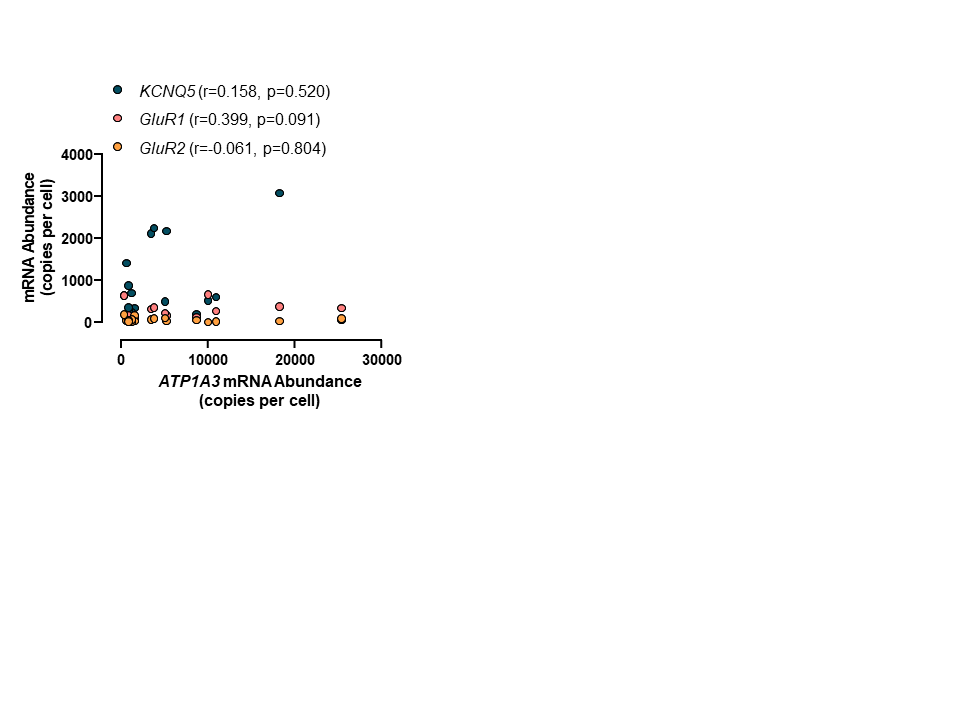

Supplement: S3 Fig — Each data point represents the mRNA expression from a single neuron (n = 19). The data underlying this figure can be found in S1 Data. (TIF) [file pbio.3001971.s003.tif]

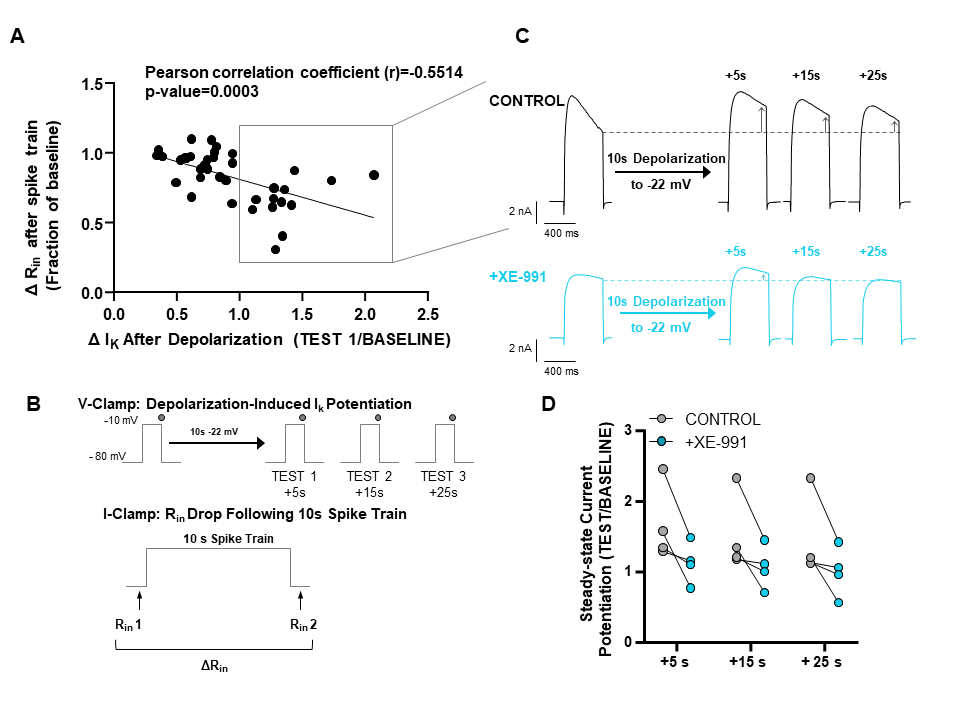

Supplement: S4 Fig — (A) Neurons with Rin decreases during the usAHP also have outward K+ currents potentiated by brief depolarization. The box highlights examples where Rin was reduced during the usAHP and outward currents were potentiated by 10 seconds of depolarization (n = 38); (B) shows the protocol to assess outward current potentiation and the activity-dependent change in Rin during the usAHP in the same neuron. The circle indicates that data are reported at the end of the step. (C) Example traces showing potentiation of the outward current by depolarization to −22 mV, which decays following stimulation over the following 10 seconds of seconds. The green trace shows the same neuron, but after the application of XE-991. XE-991 reduced the potentiation, demonstrating a role for Kv7 channels. (D) Individual data points from 4 experiments before and after application of XE-991. Each cell had potentiated outward currents that were reduced by XE-991 (n = 4). Two-way ANOVA (p = 0.0216; Drug × time interaction). The data underlying this figure can be found in S1 Data. (TIF) [file pbio.3001971.s004.tif]
