# Supplementary material for: Neuron populations use variable combinations of short-term feedback mechanisms to stabilize firing rate
Source: PLoS Biol. 2023 Jan 23;21(1):e3001971. doi: 10.1371/journal.pbio.3001971 (PMC9894548; doi:10.1371/journal.pbio.3001971)
Supplement: Attachment — Submitted filename: Pellizari et al._Response to Reviewers.docx [file pbio.3001971.s006.docx]

**Responses to Reviewers’ Comments for Manuscript # PBIOLOGY-D-22-02186R1**

We thank the reviewers for their careful and critical comments on our manuscript. We have responded to each of the points below. In addition to these responses, we have included a marked up document that notes the changes made to the original submission. These comments were taken under serious consideration and improved the quality and clarity of the paper. Below we have pasted the original comments from each reviewer. Our responses are noted in blue, and line numbers reference the marked up document. The major changes to the manuscript are as follows:

1) In response to Reviewer 1, we edited figures and revised the text.
2) In response to Reviewer 1, we added a new supplemental figure 1 showing absolute R_in_ changes during the ultraslow AHP.
3) In response to Reviewer 2, we now have clarified why we assessed single-cell mRNA for our targets of interest, as well as the interpretation of the results.
4) In response to Reviewer 2, we now present single-cell mRNA correlations with and without data points flagged as outliers by the Grubbs’ test. Regardless of whether we keep or remove outliers, the conclusions are the same.

**Reviewer #1:** Overview:  This manuscript addresses an important and fundamental problem: how neurons maintain their characteristic and fundamental patterns of electrical excitability.  This manuscript presents some novel results that are intriguing, and argue for the first time, that a population of neurons uses two different cellular mechanisms (a Na pump and a K channel), in different ratios, to achieve their characteristic activity patterns.
While the work has been cleverly and carefully done, I am always concerned by the extent to which the findings depend on the specificity of pharmacological agents.  How do we know that the drugs used are specific and used at appropriate concentrations?
The Discussion is quite thoughtful.

We thank this reviewer for their positive appraisal of our work. Below we address their specific comments, including the point regarding the specificity of the drugs we used.

Specific comments:
1.      line 34.  I don't think that the reference to Fig, 1A is needed here, and I don't like referring to figures in the Introduction.

The reference to the figure has been removed.

2.      Figure 1F. the format of these plots is a bit strange…the lines with no points make it look like there is a continuous function while there are just two points connected?  Can you plot differently?

We agree and have now plotted these data using a line to connect the “before” and “after” points for each experiment. In addition, in editing the manuscript we felt that the dark blue to represent the middle one-third of the data in Fig. 1C was hard to see, so we changed it to green.

3.      Figure 1C, I understand why these data were normalized, but isn't there a way to plot the data that doesn't lose the variance of the data by not normalizing?  Likewise Fig 1D?

Once we discovered that input resistance dropped during the ultraslow AHP (the hyperpolarization in the minute following intense stimulation), our goal became understanding the mechanisms that cause this change. The input resistance values of neurons in this population are quite variable (<https://elifesciences.org/articles/30005>, <https://www.nature.com/articles/s41598-022-15525-8>); thus, plotting the data relatively provided a convenient way to scan the data for large and small drops in input resistance. As the reviewer suggests, this was sensible based on our goal. In response to their comment regarding the absolute changes, we show the results of Fig. 1C plotted absolutely in the figure below. As you can see, we observe the same trends in the data, but it is much more difficult to get a feel for big and small changes in input resistance based on the variation in the starting value, which is the main goal of this experiment.

We have included this plot as “S1 Fig.” in the revised manuscript, as we do feel this question may arise when others are reading the manuscript. Plotting the data absolutely, we do note a trend towards lower R_in_ neurons having smaller drops in R_in_ (red) and higher input resistance cells having bigger drops in R_in_ (blue). However, the data are quite heterogeneous, and each color group does not fall nicely into high or low R_in_ categories. This is especially true for the “medium size” R_in_ drop group (green) scattered across the range in starting R_in_ values. This shows that the trends in the present manuscript are not cleanly explained by the cell’s starting input resistance. Given this variation in baseline R_in_, we do believe correlating the size of the usAHP (Fig. 1D) with the relative change in R_in_ following activity is the most sensible way to present the data and we have opened to keep the plot as it was presented in the original manuscript.

Red=Smallest Rin changes (lowest 1/3), Yellow= “medium” Rin changes (middle 1/3), Blue=Large Rin changes (top 1/3)

**B**

**a**

**s**

**e**

**l**

**i**

**n**

**e**

**5**

**s**

**1**

**0**

**s**

**1**

**5**

**s**

**2**

**0**

**s**

**2**

**5**

**s**

**3**

**0**

**s**

**3**

**5**

**s**

**4**

**0**

**s**

**0**

**200**

**400**

**600**

**800**

**Input Resistance (MΩ)**

4.      What evidence is there that XE-991 is specific for Kv7 channels?  In this species?

For XE-991, this drug blocks KCNQ/ Kv7 channels/ “m-current” in a range of species, from flies to mice (https://journals.plos.org/plosone/article?id=10.1371/journal.pone.0023898 ). Thus, we have a good deal of confidence that XE-991 blocks Kv7 in our system. Nevertheless, this reviewer’s point is well taken, and with a bit of searching, we did not find any articles that specifically used XE-991 in this species (which we admittedly found surprising as the “m-current” was discovered in bullfrog sympathetic ganglia neurons). To enhance our confidence in this drug’s action we performed pilot experiments that better isolate Kv7 channel function from the total outward current and determined its sensitivity to XE-991. The data are shown on the next page.

Kv7 is a non-inactivating current that slowly deactivates upon repolarization of the membrane (<https://www.science.org/doi/10.1126/science.282.5395.1890>). Thus, we performed two pilot voltage clamp experiments that activated the Kv7 currents by a step from -68 to -32 mV and then deactivated it by repolarizing the membrane to a step to -52 mV. This leads to a deactivating tail current that lasts >100 ms representing the channel slowly closing upon repolarization (black trace in both experiments). When we bath applied XE-991, the deactivating tail current was mostly abolished (gray trace in both experiments). These recordings show that XE-991 blocks a non-inactivating, slowly deactivating outward current, consistent with this drug’s action on Kv7 channels.

To the question of specificity, we searched the literature for known off-target effects of XE-991. The only published example we found that could confound our interpretation was a block of ERG K^+^ channels (<https://onlinelibrary.wiley.com/doi/full/10.1111/j.1742-7843.2007.00048.x>). However, the EC50 of XE-991 on these channels is >10x the concentration we used (100 uM), while the concentration of XE-991 in our study (10 uM) should block ~80-90% of the Kv7 current (<https://www.science.org/doi/10.1126/science.282.5395.1890>). Although we cannot possibly rule out all potential off-target effects (which is true of any pharmacological or genetic approach), we do believe the chosen dose of XE-911 blocked Kv7 channels and did so while minimally inhibiting ERG channels.

**Example 2**

200 ms

100 pA

-32 mV

-62 mV

Control

+XE-991

-32 mV

-62 mV

**Example 1**

200

pA

Control

+XE-991

200 ms

Although ouabain (inhibitor of the Na^+^/K^+^ ATPase) was not specifically questioned by the reviewer, we wanted to address that our concentrations are consistent with use in amphibians (<https://www.sciencedirect.com/science/article/pii/S0960982222000239>). Specifically, we show that 2 µM ouabain blocks the activity-dependent form of the pump. That is, it blocks hyperpolarization caused by activity but does not influence the resting membrane potential. In contrast, 20 µM ouabain appears to block the constitutively active form of the pump, as this dose causes a rapid and consistent depolarization of the resting membrane potential. These responses are consistent with the high ouabain affinity of the low Na+ affinity form of the pump and lower ouabain affinity of the higher Na+ affinity form of the pump (<https://www.imrpress.com/journal/FBL/10/3/10.2741/1704>).

Reviewer #1 Cross Comments:
I couldn't get back to the original manuscript, so I can't see what references the other reviewer was arguing about. More critically, I sort of doubt that this idea was already in the literature, and I don't understand the other reviewer's minor comment #3. I still think the paper is quite intriguing and original and think you should give the authors the chance to revise it.

We thank this reviewer for their support of our study.

**Reviewer #2:** This interesting manuscript studies regulation of the breathing related activity of vagal motoneurons in an amphibian model. The goal was to evaluate the role of activity-dependent regulation of network bursting activity by the Na+/K+ pump and voltage-gated K+ channels (Kv7). The key finding is that a given vagal motoneuron has differing levels of pump activity and Kv7 channels. The methods are straightforward, the paper is mostly well written, and the results support the concept of differential molecular controllers of membrane excitability.

We thank this reviewer for a critical review of our manuscript and for the valuable feedback.

Major concerns.

There are some important weaknesses. First, the idea is not new. More exhaustive work on this problem has been published based on the recording of both lumbar motoneurons (your reference 17) and preBotzinger complex inspiratory interneurons (Krey et al., Front Neural Circuits. 2010 Nov 29; 4:124), which is not cited.

We want to make it clear that the manuscript did not present any arguments about novelty regarding the role of the Na^+^ pump for the regulation of neuronal output. This is well-established and formed the rationale for our study, so we agree it is not novel. Lots of the literature pointed to the dynamic Na^+^ pump as a mechanism that integrates neuronal activity over the time course of around a minute in motor systems, which quickly modulates excitability to control neuronal output through feedback homeostasis. Thus, when we began these experiments intending to study how the Na^+^ pump dynamically regulates membrane excitability in our system, we were stuck that the classic signature of “Na^+^ pump feedback” (the ultraslow AHP) was accompanied by graded changes in input resistance. This was not consistent with the exclusive function of an electrogenic Na^+^ pump. Thus, we tracked down the relevant K^+^ channel that was rapidly potentiated by membrane depolarization over the same timescale and uncovered a new organizational strategy at play between these two feedback mechanisms. These results led us to the new finding that neuronal populations can inversely scale two different activity regulators across cells to generate their characteristic firing outputs.

The manuscript also makes no claims about novelty regarding the use of two (or more) mechanisms to do anything in neurobiology, or biology in general. However, we know of no other examples where multiple mechanisms for activity-dependent feedback are coordinated inversely in a graded way across a neuronal population, with each mechanism recruited by a different “read out” of activity (intracellular Na+ engaging the pump and depolarization potentiating Kv7 currents for tens of seconds). The field of firing rate homeostasis was founded on (and continues to be dominated by) the straightforward idea that a single “read out” of activity is critical for the regulation of firing rate within a given cell type and timescale (e.g., intracellular Ca^2+^). In contrast, our results provide strong evidence that every neuron in a population may use its own “signature” feedback solution, sensing different aspects of activity compared to its neighboring neurons to generate stable firing rates. This diverges significantly from the vast majority studies addressing how neurons regulate their firing rates.

We acknowledge this pump can participate in many aspects of neuronal function, and we aimed to cite papers in the introduction where it was playing a clear role in activity-dependent feedback homeostasis. We did not include the Krey et al. article because, based on our interpretation of those data, it seems to be playing at least some role in setting the resting membrane potential, as they observed the same response we see after we inhibit the constitutively active Na^+^ pump (slow depolarization of the resting membrane potential consistent with the loss of ion gradients; Fig. 1A of the Krey study). This makes it difficult to assess whether effects they observe are to due activity-dependent feedback (as we isolated with 2 uM ouabain) or through disrupting ion gradients by way of blocking the “housekeeper” form of the Na^+^ pump (which we inhibited with 20 uM ouabain as a control to ensure 2 uM ouabain predominately inhibited the activity-dependent isoform). Further, the Krey paper does not show the Na^+^ pump playing a role in the “ultraslow AHP” (a hyperpolarization following burst firing that lasts for upwards of 1 minute), which differs from what we studied in the present manuscript. We edited the entire manuscript to ensure it is clear we are studying the “dynamic” form of the Na^+^ pump.

Second, almost all results are based on correlations, with many of the relations relying on relatively small sample sizes. For example, the data in Fig. 3C seems to be heavily influenced by two data points in the upper right quadrant of the graph; what is the correlation coefficient without these two data points? I do understand that short reports do not require a full data set, but novelty needs to be made crystal clear.

Using correlations is the “gold-standard” for understanding co-expression relationships between different ionic currents. This is how we were able to uncover the reciprocal relationship between the dynamic Na^+^ pump and Kv7 channels. Our sample sizes used to make these arguments fall within (or exceed) that which is consistent for those using biological neurons (n=12 to 43 in our study; for some literature examples see Schulz et al., 2006, *Nature Neuroscience*, <https://www.nature.com/articles/nn1639>, Temporal et al. 2012, *J Neurophysiol*., <https://journals.physiology.org/doi/full/10.1152/jn.00622.2011>, Temporal et al., 2014, *Current Biology*, https://www.sciencedirect.com/science/article/pii/S0960982214007842#fig1, Ransdell et al., 2012, *J. Neurosci*., <https://www.jneurosci.org/content/32/28/9649.short>).

For the single-cell mRNA data in question (Fig. 3C) we agree the scatter is quite variable, which is consistent which mRNA expression in single neurons. However, we disagree with somewhat arbitrarily defining data points as outliers by eye. Thus, we ran an outlier test (Grubbs’ test, α=0.01) to statistically assess this data set for outliers. This analysis flagged only one data point as an outlier (the highest data point in the KCNQ2 data set). When we rerun the correlation analysis, there remains a significant correlation between KCNQ2 and α3 subunit of the Na^+^ pump (p=0.0144). In addition, if we rerun the outlier test with a less stringent α value of 0.05, the test now flags the highest point in each group as outliers. When we rerun the correlation analysis for KCNQ3 without its highest data point, the correlation is still significant (p=0.0035).

Strengthening our conclusions that these correlations are real and specific to KCNQ2-KCNQ3 and the α3 subunit of the Na^+^ pump (Fig. 3C), we did not observe the same correlation between KCNQ5 and α3 subunit of the Na^+^ pump, as well as between two AMPA-glutamate receptor genes and α3 subunit of the Na^+^ pump (shown in Supplemental Fig. 3). These “negative” results serve as controls and give us confidence in the relationships that were significant in Fig. 3C.

All this said, we agree with this reviewer and are sensitive to the issues that outliers can cause in correlation analyses. Therefore, we now present these p values, regression lines, and slopes with (solid) and without (dashed) the highest data point in the data set in Fig. 3C of the revised manuscript, which were flagged as outliers in the Grubbs’ test.

Third, the paper lacks discussion of how this mechanism would work to control the network's cycle period to affect an increase or decrease in ventilatory output.

Thank you for encouraging us to discuss how these mechanisms may be implemented in vivo. Our original manuscript discussed the potential for why multiple combinations of different activity controllers may be adaptive (lines 237-250). We have now added text that indicates we believe this strategy acts to constrain excitability of the motor pool in an ongoing manner (lines 231-233). **“Each neuron in the vagal motor pool appears to continuously sense different aspect of its activity, and in different weights relative to neighboring neurons, to constrain population motor output for breathing.”** Functionally, we believe these mechanisms in motoneurons would prevent the animal from taking “too big” of a breath by keeping motoneurons from generating excessive firing (Fig. 4). Again, and importantly, each neurons appears to be sensing intracellular Na^+^ and mean membrane voltage in varying amounts depending on its unique ratio of Na^+^ pump and Kv7 channel densities to accomplish this goal. Given that these are motoneurons driven by the presynaptic rhythm generating neurons, we make no claims that these processes influence cycle period, as this aspect of network function would be most influenced by the pace of the rhythm generating network which we did not study here.

Minor Concerns.

1.      The mRNA results are confusing and insufficiently discussed and interpreted.

Correlated ionic currents often have a transcriptional basis, whereby correlated mRNAs give rise to those ionic current relationships. We agree that an inverse relationship between function and a positive relationship between mRNAs that code for these proteins is not straightforward. We have edited lines 103-104 to better describe (1) why we measured single cell mRNA and lines 204-230 (2) to more clearly interpret the discrepancy between mRNA and protein function within the context of the field. In response to the comment above, we have also reanalyzed the data and presented these results with and without outliers identified by the Grubbs’ test in Fig. 3C.

2.      Lines 148-150. The figures appear to be mis-labeled.

Thank you for the careful read and catching this error. We have corrected it.

3.      The simulated synaptic input studies are a nice addition, though it should be mentioned that even though this may be better than bath-application of drugs (Line 143), changes in voltage-dependent ion channels cannot be avoided.

We want to clarify the experiment in question. We did bath apply drugs to the neurons in the brain slice that were receiving simulated synaptic input. Although we show evidence of slow activity-dependent feedback in these motoneurons receiving real synaptic input in the intact network (Fig. 4A), our intent with this approach was to avoid confounds with bath applying drugs to the entire network that might alter activity of the rhythm generating circuits that give rise to synaptic drive onto the motoneuron. By bath applying drugs to slices and injecting simulated synaptic currents, we could restore the “normal” synaptic activity these neurons receive without disrupting the activity of the neurons that cause that synaptic input. We are aware of the limitations of using a pharmacological approach to address this question. Please see our response to Review #1 regarding the ability of these drugs to block their intended targets and the specificity of this block in our system.

Reviewer 2 Cross Comments:
I remain unconvinced that the idea is brand new but asked an extremely bright research Asst. Professor in the lab to also have a look as well. Their comments:

I found the manuscript interesting and the experiments were carefully designed, however I don’t think that the results are significant enough to be published in PLOS Biology short reports.

The novelty of these results is definitely questionable. There are many publications citing multiple mechanisms within one neuron type that underly slow afterhyperpolarization.

We want to make it clear that this paper does not address the slow afterhyperpolarization (which happens over several hundred milliseconds). This paper addresses the “ultraslow afterhyperpolarization,” which has emerged as a way for neurons to integrate activity and regulate neuronal output over the timescale of around a minute. The vast majority of the work on the ultraslow afterhyperpolarization as it relates to the control of motor systems has shown an apparently exclusive role for the “dynamic” Na^+^ pump (Zhang & Sillar, 2012, *Current Biology*, <https://www.sciencedirect.com/science/article/pii/S0960982212000887>, Hachoumi et al., 2021, *Current Biology* <https://www.sciencedirect.com/science/article/pii/S0960982222000239>, Picton et al., 2017, *J. Neurosci*., <https://www.jneurosci.org/content/37/4/906.abstract>, Pulver & Griffeth, 2009, *Nature Neuroscience*, https://www.nature.com/articles/nn.2444). Instead, we show that the Na^+^ activation of the pump and rapid potentiation of Kv7 channels through a depolarization-sensitive mechanism (Supplemental Fig. 4) combine in graded way from cell to cell to give each neuron in a population its own unique solution for firing rate regulation over the ~minute timescale. We believe this organizational strategy for two feedback mechanisms is novel and has not been demonstrated in the literature.

We agree that different authors have assessed the Na^+^ pump and Kv7 channels within the same neuronal population, just as many authors have studied many of different proteins and cellular mechanisms in the same cell type. Yet, our work is the first of our knowledge to show that there is a seemingly precise strategy at play that scales these two mechanisms in a graded manner across cells to give rise to firing rate homeostasis. As we describe in the next comment, this appears to be a previously unappreciated way for neuronal populations to control their firing rate through activity-dependent feedback, which we interpret as a novel insight.

The one potentially novel piece is the finding that motoneurons have graded expression of the two mechanisms described, i.e if Na+ pump is high then Kv7 is low and vice versa, but that seems intuitive given the remarks in the introduction that neurons have a “dominant” sensory signal for a given regulated variable.

We centered our conclusions around this point, and we agree this is the most critical insight. Yet, we disagree that it is intuitive. Most of the field of neuronal regulation works under the assumption that neurons track a key feature of their activity over a particular time scale to regulate intrinsic excitability (e.g., Ca^2+^, Na^+^, etc.). The work we cited in the introduction makes this assumption, as well as a vast literature on “homeostatic plasticity” that occurs over longer timescales. We did not find experimental studies making a “multiple activity sensor” assumption for the regulation of intrinsic excitability. This framework appears to have arisen from decades of modeling work, where model neurons must track one key “activity signal” because it is difficult to implement multiple activity sensors while achieving stable neuronal output (see here for one example that articulates the problem, O’leary et al., *Neuron*, 2014; <https://www.sciencedirect.com/science/article/pii/S089662731400292X>). Thus, the fact that we find two different “activity sensors” are scaled reciprocally across neurons means that some neurons are tracking their activity through one kind of “activity signal” (e.g., Na^+^ through the pump), others follow a different signal (mean depolarization of the membrane through rapid Kv7 potentiation), as well as a large range of combinations in between these two extremes (see schematic above). This “graded” strategy for organizing homeostatic control mechanisms is captured by our physiology experiments and presents an example that stands in contrast to a very large body of work on how feedback control gives rise to stable neuronal output.

In addition, as written, the authors seem to suggest that Na+ pump and Kv7 are the only two mechanisms by which these neurons maintain their firing rates. I don’t feel like they performed a thorough investigation of other possible mechanisms and, therefore, the idea that the gradation of these two specific channels is the sole mechanisms is overstated.

We agree that there are probably more feedback mechanisms at play in these neurons. Lines 98-99 of the Results read, **“Some cells exhibited a residual usAHP after the application of both ouabain and XE-991, suggesting that additional feedback processes exist in some neurons.”** Thus, we acknowledged that more complexity probably exists than we fully flesh out here. Nevertheless, additional regulatory mechanisms that may exist do not detract from the intriguing relationship we identified in the present study. We are interested in how neurons might integrate even more cellular reads outs of activity and look forward to pursuing this in future studies.

Finally, the PCR data doesn’t add value. In my opinion, imunohistochemistry showing gradation of these two channels across this neuron
population would have been better to support for their ephys data and would make the manuscript stronger, but without this, what is left is a lovely, yet not groundbreaking, ephys data set.

In response to previous comment regarding single-cell mRNA, we have tried to better contextualize why we measured single-cell mRNA levels for these two mechanisms. Namely, co-expression of ionic currents at the functional level appears to be “set in place” by transcriptional mechanisms that correlate mRNAs. In addition, we gave some further explanation regarding the interpretation of these results in the text. We agree that immunohistochemistry would be an excellent addition! However, there are no specific antibodies for these proteins subtype in our species. Thus, at this time, this technique seems out of reach for our species, but we are highly interested in developing new tools to address these critical questions in the future.
